# Exploring Antiurolithic Effects of Gokshuradi Polyherbal Ayurvedic Formulation in Ethylene-Glycol-Induced Urolithic Rats

**DOI:** 10.1155/2013/763720

**Published:** 2013-03-11

**Authors:** Amol L. Shirfule, Venkatesh Racharla, S. S. Y. H. Qadri, Arjun L. Khandare

**Affiliations:** ^1^Food and Drug Toxicology Research Centre, National Institute of Nutrition, Hyderabad 500007, India; ^2^Department of Pharmacology, CMR College of Pharmacy, Hyderabad 500007, India; ^3^Pathology Division, National Institute of Nutrition, Hyderabad 500007, India

## Abstract

*Gokshuradi Yog (GY)* is a polyherbal ayurvedic formulation used traditionally for several decades in India for the treatment of urolithiasis. The aim of the present study was to determine the underlying mechanism of *GY* action in the management of calcium oxalate urolithiasis. The effect of *Gokshuradi polyherbal aqueous extracts* (*GPAEs*) was studied on various biochemical parameters involved in calcium oxalate formation by employing *in vitro* and *in vivo* methods. *GPAE* exhibited significant antioxidant activity against 1, 1-diphenyl-2-picrylhydrazyl free radical and inhibited lipid peroxidation in the *in vitro* experiments. The rat model of urolithiasis induced by 0.75% ethylene glycol (EG) and 1% ammonium chloride (AC) in water caused polyuria, weight loss, impairment of renal function, and oxidative stress and decreased antioxidant enzyme activities in untreated control groups. However, *GPAE-* (25, 50, and 100 mg/kg) treated groups caused diuresis accompanied by a saluretic effect and revealed significant increase in antioxidant enzyme activities along with decreased oxalate synthesizing biochemical parameters at higher doses. This study revealed the antiurolithic effect of *GPAE* mediated possibly through inhibiting biochemical parameters involved in calcium oxalate formation, along with its diuretic and antioxidant effects, hence supporting its use in the treatment of calcium oxalate urolithiasis.

## 1. Introduction

Calcium oxalate urolithiasis has become a rising problem in many countries due to geographical/genetic variations and modern lifestyles. People living in drought and arid area suffer more from intestinal hyper absorption of calcium oxalate leading to kidney stones. It is estimated that 12% of the world population is affected by kidney stones with a recurrence rate from 70 to 80% in males and from 47 to 60% in females [1, 2]. Calcium containing stones are the most common comprising about 75% of all urinary calculi, which are observed in the form of pure calcium oxalate (50%) or calcium phosphate (5%) and a mixture of both (45%). Many factors affect the growth of urinary calculi in which mineral metabolism plays an important role [3].

The treatment modalities like surgery and drug therapy are practiced in the management of kidney stones but have some limitations. Surgical procedures like (extracorporeal shock wave lithotripsy) ESWL have increased risk of stone recurrence and are also not affordable to poor sufferers. The armamentarium of therapeutic agents also does not have any effective drugs except for some diuretics like thiazides and furosemide. This warranted a search for new drug therapy which will be cost effective and can target multiple etiological risk factors in urolithiasis. Ayurveda, a classical Indian authentic literature, reported the traditional use of *Gokshuradi polyherbal formulation* (*GPF*) and is being practiced since many years in the management of kidney stones. In our preliminary clinical studies, *GPF* has revealed expulsion of kidney stones in naturally occurred urolithic patients which was confirmed by X-ray analysis conducted before and after four weeks of treatment [4]. *GPF* consists of parts of five different medicinal plants that is, dry fruits of *Tribulus terrestris *L. seeds of* Hygrophila spinosa *T. Anders, and roots of *Ricinnus communis *L,* Solanum anguivi* Lamk. and *Solanum surattense *Burm. *GPF* decoction was prepared by mixing the fine powder of these individual parts at equal proportion as mentioned in the ayurvedic literature and then it was administered to the patients. 

The individual component plants of *GPF* are known to possess various pharmacological activities. In Ayurvedic medicine, the fruits of *T. terrestris* L. are recommended for the treatment of urinary disorders erectile dysfunction, and in traditional Chinese medicine, it has been used as an antihypertensive in coronary heart disease [5]. It also stimulates melanocyte proliferation and therefore is a putative treatment for Vitiligo along with its known antibacterial and cytotoxic activities [6, 7]. *H. spinosa* T. Anders, another component of this formulation has been used in Ayurveda for various ailments like jaundice, hepatic obstruction, rheumatism, inflammation, pain, diuretic, and aphrodisiac and in the treatment of urinary disorders [8, 9]. This plant is known to possess hypoglycemic activity in human subjects [10], antitumor [11], haematinic [12], antinociceptive [13], hepatoprotective [14, 15], free radical scavenging, and lipid peroxidation activities [16]. *R. communis *stems have anticancer, antidiabetic, and antiprotozoal activity [17]. *Solanum anguivi* (Lam.), also known as *Solanum indicum* sp. distichum (Schumach. and Thonn.), is cultivated for culinary purposes in many parts of Africa, India, and the Arab Peninsula. The fruits of this plant are used as nutritious vegetables on account of their high content of starch, calcium, vitamin A, ascorbic acid, and phosphate. *Solanum xanthocarpum *(SX) Schrad. & Wendl. (Family: Solanaceae) is commonly known as the Indian night shade or Yellow berried night shade found throughout India, mostly in dry places as a weed along roadsides and waste lands [18]. It has held a place of some importance in the Indian *Materia Medica,* primarily as an expectorant and antipyretic. Various medicinal properties are attributed to it, particularly in the treatment of asthma, chronic cough, and catarrhal fever [19]. It is one of the members of the *dashamula* (ten roots) of the Ayurveda [20]. In Ayurveda, this plant is described as pungent, bitter, digestive, and alternative astringent. Its stems, flowers, and fruits are bitter, carminative. Root decoction is used as febrifuge, effective diuretic and expectorant [21].

Although, *GPF* is well recognized in Indian traditional medicine as having antiurolithic effect, but no scientific data have been published supporting its underlying mechanism of action on calcium oxalate stones. Therefore this study was proposed to determine the therapeutic potential and mechanism of action of aqueous extracts from this formulation in the management of urolithiasis by employing *in vitro* and *in vivo* methods.

## 2. Material and Methods

### 2.1. Chemicals and Reagents

All the chemicals used were of analytical grade. Ethylene glycol, thymol, reduced glutathione, 5-5′-dithiobis, 2-nitrobenzoic acid, thiobarbituric acid, furosemide, H_2_O_2_, trichloroacetic acid, 1,1,3,3,-tetraethoxypropane, and guanidine hydrochloride were obtained from Sigma Chemical Company, St. Louis, MO, USA. Reagents used for histological preparations were eosin spirit soluble, hematoxylin, and xylene from Himedia Chemical Limited, Bombay, India. Kits used in this study for the determination of calcium, magnesium, blood urea nitrogen (BUN), creatinine, superoxide dismutase, glutathione peroxidase, oxalate, and citrate were purchased from Spinreact, Germany, and Ambika diagnostics, India.

### 2.2. Plant Materials

Plants used for the formulation were collected from local forests of Nanded District, Maharashtra, India, and authenticated by experts. Voucher specimens were deposited in the herbarium of the School of Life Sciences, S.R.T.M. University, Maharashtra, India. Respective parts of each plant as mentioned previously were collected and then shade dried. Dried parts were cleansed of extraneous matter and then grounded to fine powder in a grinder.

### 2.3. Standard Drug

Cystone polyherbal formulation from the Himalaya Drug Co., Bangalore, India was used as a standard drug in this study. The formulation contained *Didymocarpus pedicellata*, *Saxifraga ligulata*, *Rubia cordifolia*, *Cyperus scariosus*, *Achyranthes aspera*, *Onosma bracteatum*, *Vernonia cinerea*, purified* Shilajeet*, and Hajrul Yahood Bhasma.

### 2.4. *In Vitro* Antioxidant Effect

Antioxidant effect of *GPAE* was determined by free radical scavenging activity and lipid peroxidation inhibitory effects. 0.1 mM solution of DPPH radical in methanol was prepared, and 1 mL of this solution was added to 3 mL of *GPAE* at different concentrations to determine the free radical scavenging activity [22]. Solutions were incubated for 30 min at room temperature, and then absorbance was measured at 517 nm. Decrease in DPPH solution absorbance was considered as an increase of the DPPH radical-scavenging activity. This activity is given as % DPPH radical scavenging which was calculated in the equation using DPPH solution as control.

Lipid peroxidation inhibitory activity was determined in the isolated rat kidneys which were electrically homogenized in ice cold 50 mM phosphate buffer saline (PBS) and adjusted to pH 7.4. The homogenate was processed further, and lipid peroxidation inhibitory activity was determined by a reported method [23]. The inhibition ratio was calculated using the formula given for free radical scavenging activity.

### 2.5. *In Vivo* Studies

#### 2.5.1. Animals

The animal care and handling was in accordance with the internationally accepted standard guidelines for use of animals, and the protocol was approved by institutional animal ethical committee (IAEC number P25/7-2011/GBR) at the National Institute of Nutrition, Hyderabad, India. Wistar rats (180–220 g) of either sex used for this study were sourced and housed at the National Centre for Laboratory Animal Sciences, Hyderabad, India, and kept in plastic cages (47 cm × 34 cm × 18 cm) with saw dust (renewed after every 48 h), under a controlled temperature of 23–25°C and 12 h light-dark cycle. Animals were given standard rat chow diet available at the centre. Animals had free access to food and water *ad libitum* throughout the study except 24 h before and during 6 h of diuretic study, and while collecting 24 h urine samples, food was withdrawn.

#### 2.5.2. Preparation of *GPAE* and Cystone for Gastric Feeding

Initially, *GPF* was prepared by mixing equally the respective parts of each plant at a ratio of 1 : 1 : 1 : 1 : 1, respectively. In order to prepare the decoction for gastric gavages, 100 g of *GPF* and 500 mL of doubly distilled (dd) water were heated in an autoclave for 15 min at 121°C. After the boiling and sterilization procedure, the powder turned into a jelly-like paste which was dissolved in dd water to reach a final volume of 750 mL. Then, the solution was stored at 4°C for 7 days. Subsequently, the solution was poured out and centrifuged at a rate of 1,500 rpm for 10 min. Finally, the *GPAE* feeding solution containing 50 mg of formulation per milliliter was prepared. The same procedure was performed for Cystone to make 100 mg per milliliter of solution.

#### 2.5.3. Diuretic Activity of *GPAE*

The diuretic activity of *GPAE* was studied on Wistar rats of either sex (180–220 g) as described previously [24]. Animals were divided with matched body weight and sex into groups of 6 animals each. Negative and standard drug control groups were given saline by gavage (20 mL/kg) and Furosemide (10 mg/kg of body weight), respectively. The rest of the groups were given different doses of the *GPAE* (25, 50, and 100 mg/kg) dissolved in saline. Subsequently, the animals were placed individually in metabolic and diuretic cages. The urine was collected, in graduated cylinders for 6 h at 1 h intervals. Total urine excreted out was collected and the volume was determined. The pH of the pooled urine from each animal was determined by using pH meter, Na^+^ and K^+^ concentrations on flame photometer, and Ca^2+^ concentration by using commercially available kits.

#### 2.5.4. Effect of *GPAE* and Cystone on Animal Model of Urolithiasis

Antiurolithic activity of *GPAE* and Cystone was determined in an animal model of calcium oxalate urolithiasis as described by Atmani et al. [25]. The dose which had caused the significant increase in urine output in diuresis study was selected for this study. Twenty-four male Wistar rats (weighing 180–220 g) were divided with matched body weights into 5 groups of 6 animals each, which were then randomly selected to receive various treatments as follows.

Group I rats, served as vehicle-treated control, received intraperitoneal (i.p.) injections of normal saline (2.5 mL) once in 24 h and water *ad libitum *daily for 21 days (normal control).

Group II rats received stone-inducing treatment for 21 days, comprised of 0.75% (w/v) EG with 1% (w/v) ammonium chloride for 5 days; following this the water supply was switched to 0.75% EG alone in water, along with saline treatment (positive control urolithic group).

Group III rats received *GPAE* (50 mg/kg) through gastric gavages and simultaneously received stone-inducing treatment similar to the positive control daily for 21 days (treatment group, low dose).

Group IV rats received *GPAE* (100 mg/kg) through gastric gavages and simultaneously received stone-inducing treatment similar to the positive control daily for 21 days (treatment group, high dose).

Group V rats received standard drug-Cystone, (100 mg/kg) through gastric gavages and simultaneously received stone-inducing treatment similar to the positive control daily for 21 days (treatment group, standard drug).

#### 2.5.5. Biochemical Examination

Immediately before and at the end of a total 21 days of treatment, 24 h urine samples were collected in the presence of a few thymol crystals, by housing animals individually in the metabolic and diuretic cages. Water intake was also determined simultaneously. Following volume and pH determination, part of each 24 h urine sample was acidified to pH 2 with 5 M HCl. Both acidified and nonacidified urine samples were then centrifuged at 1500 ×g for 10 min to remove debris and supernatants were stored at −20°C until analyzed. In acidified urine samples, oxalate, calcium (Ca^2+^) and magnesium (Mg^2+^) contents were determined by using commercially available kits, while inorganic phosphate excretion was determined by reported method [26]. In nonacidified urine samples, citrate, creatinine, and uric acid, while in serum, creatinine and BUN were estimated with the help of kit-based methods.

Blood was collected through cardiac puncture from animals under ether anesthesia, and consequently both the kidneys and liver were excised. Animals were then sacrificed by CO_2_ inhalation.

#### 2.5.6. Histopathological Examination

Organs were rinsed in ice cold physiological saline and weighed. The right kidney was fixed in 10% neutral buffered formalin, processed in series of graded alcohol and xylene, embedded in paraffin wax, sectioned at 5 *μ*m, and stained with Haematoxylin and Eosin for examination under polarized light microscope. To count the number of crystalline deposits, a field of 100x was then randomly selected from each region, and calcium oxalate deposits were counted. The total number of deposits in each specimen was reported, and calcium content in kidney tissues was determined by atomic absorption spectrophotometer.

#### 2.5.7. Lipid Peroxidation Inhibitory Effect

The left kidney was worked into 10% homogenate in PBS (50 mM, pH 7.4), centrifuged at 1500 ×g, and the supernatants were used to assess various antioxidant and oxalate forming enzymes activities and biochemical parameters like reduced glutathione (GSH), malondialdehyde (MDA), and protein carbonyl content.

Kidney homogenates were estimated for MDA content by thiobarbituric acid reactive method [27]. The protein carbonyl content was estimated by the protein derivatization with dinitrophenyl hydrazine (DNPH) into chromophoric dinitrophenyl hydrazones by the method of Levine et al. [28], and the carbonyl content was calculated using the DNPH molar extinction coefficient of 22,000 M^−1^ cm^−1^. GSH was estimated as total nonprotein thiol (SH) group following the method described by Moron et al. [29]. Superoxide dismutase (SOD) and glutathione peroxidase (GPx) were determined by using commercially available kits. Catalase activity was determined by monitoring the decomposition of H_2_O_2_ at 240 nm with a spectrophotometer [30]. Catalase activity was calculated by using *ε*_240_ (molar extinction coefficient) = 0.0394 mmol^−1^ min^−1^ for H_2_O_2_ and expressed as *μ*moles of H_2_O_2_ decomposed per min under standard conditions at 25°C. Glycolate oxidase (GOx) activity in liver and lactate dehydrogenase (LDH) in both liver and kidney were determined by reported method [31, 32].

#### 2.5.8. Statistical Analysis

The data expressed are mean ± standard error of mean (S.E.M.) and the median inhibitory concentration (IC_50_ value) with 95% confidence intervals. All statistical comparisons between the groups are made by means of one way analysis of variance with post hoc Student's *t*-test. The *P*  values less than 0.05 are regarded as significant. The concentration-response curves were analyzed by nonlinear regression using Graph Pad Prism (Graph Pad Software, San Diego, CA, USA).

## 3. Result

### 3.1. *In Vitro* Studies

#### 3.1.1. Antioxidant Effect

**Figure 1:**
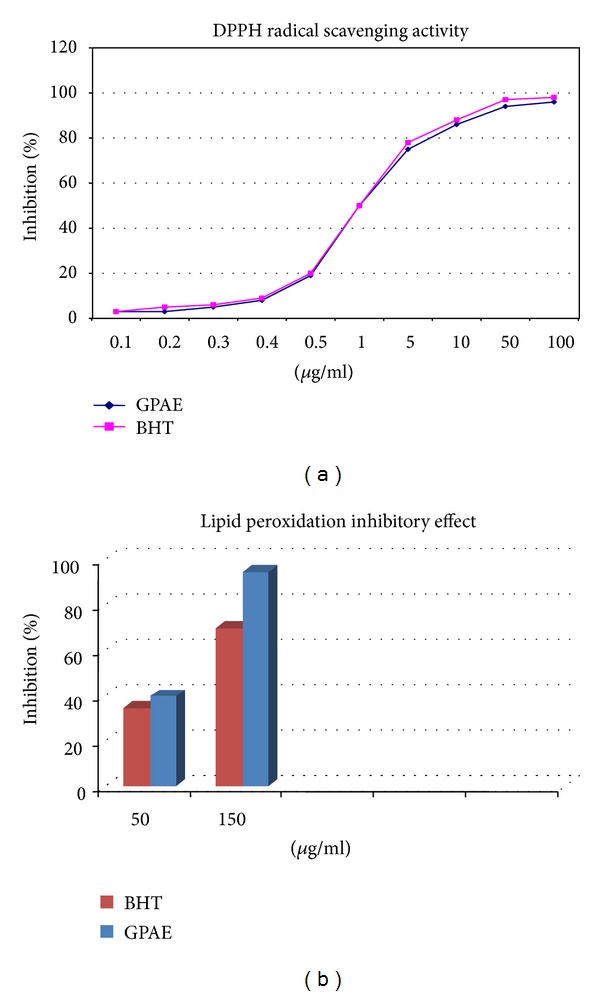
(a) DPPH radical scavenging activity. (b) Lipid peroxidation inhibitory effect of *GPAE* and the reference drug butylated hydroxytoluene (BHT). Symbols shown are mean ± S.E.M. (*n* = 3).

*GPAE *revealed DPPH radical scavenging activity with IC_50_ value of 2.5 (1.05–2.90) *μ*g/mL (Figure 1(a)) and inhibited lipid peroxidation of the rat kidney homogenate *in vitro* by 36.37 ± 1.86 and 68.72 ± 0.25% at 50 and 150 *μ*g/mL, respectively (Figure 1(b)). The standard chemical, BHT, similarly showed DPPH radical scavenging activity with IC_50_ value of 2.56 (1.02–2.85) *μ*g/mL and inhibited *in vitro* lipid peroxidation by 38.4 ± 1.22 and 94.5 ± 4.6% at 50 and 150 *μ*g/mL, respectively.

### 3.2. *In Vivo* Studies

#### 3.2.1. Diuretic Effect

The effect of various doses of *GPAE* on rat urine volume, pH, and Na^+^, K^+^, and Ca^2+^ excretion is given in Table 1. *GPAE* increased urine volume at 50 mg/kg (*P* < 0.05) and 100 mg/kg (*P* < 0.01) indicating diuretic effect. At the next higher dose (200 mg/kg), urine volume did not increase above that of saline-treated group (*P* > 0.05). The reference diuretic drug, Furosemide at 10 mg/kg, showed statistically significant increase in the urine output (*P* < 0.01). In addition to an increase in the urine output, *GPAE*, and similarly Furosemide, increased urine excretion of Na^+^ and K^+^ compared to the saline-treated control animals at higher dose. *GPAE* caused significant increase in the urine pH and decrease in Ca^2+^ excretion (*P* < 0.01) at 100 mg/kg comparable to the standard drug, Furosemide (10 mg/kg).

#### 3.2.2. Effect Observed in the Animal Model

The baseline parameters recorded before the start of treatment like body weights, water intake along with urine volume, pH, and composition were not significantly different among all the groups. The parameters recorded after the treatment are given in Table 2. As compared to normal control animals, water intake and 24 h urine volume were high in the urolithic group (*P* < 0.01). Stone-inducing treatment also reduced pH of urine in the urolithic group as compared to that of the normal control group, although not to a significant extent. Simultaneous treatment with *GPAE* at 100 mg/kg in treatment groups reduced the increase in urine volume and water intake (*P* < 0.05) as compared with the control animals (*P* < 0.05). There was an increased oxalate and decreased Ca^2+^ concentration (*P* < 0.01) of the urine collected from the urolithic animals as compared to normal control. *GPAE* and Cystone groups exactly reversed this effect, that is, decreased the urinary oxalate (*P* < 0.01) and increased Ca^2+^ (*P* < 0.05) excretion at all the treated doses and remained significant at 100 mg/kg in *GPAE*-treated group. Other changes in the urine composition like decreased levels of citrate and Mg^2+^ and increased excretion of uric acid and inorganic phosphate in urolithic group were not statistically significant. *GPAE-* and Cystone-treated groups also did not show any statistically significant change in these parameters and remained comparable to that of control animals. The urolithic treatment revealed loss in body weight of the urolithic animals (*P* < 0.01), whereas *GPAE-* and Cystone-treated animals showed net gain in the body weights at all the tested doses. Kidneys of urolithic group animals were heavier than those the control animals (*P* < 0.01), whereas *GPAE-* (100 mg/kg) treated rat kidneys did not show any significant increase in weight as compared with control; however the *GPAE-* (50 mg/kg) and Cystone- (100 mg/kg) treated animal's kidneys were slightly heavier than those the control animals (Table 4).

Urolithic treatment was found to disturb renal function in urolithic animals as evident from the increased BUN and serum creatinine and reduced creatinine clearance. *GPAE*-treated groups decreased the serum creatinine and BUN levels at 100 mg/kg, and similar observations were made in the Cystone-treated groups at 100 mg/kg as compared to control. The creatinine clearance was significantly increased at 100 mg/kg in *GPAE*-treated groups as compared to urolithic animals but was not significant at 50 mg/kg (Table 3).

**Figure 2:**
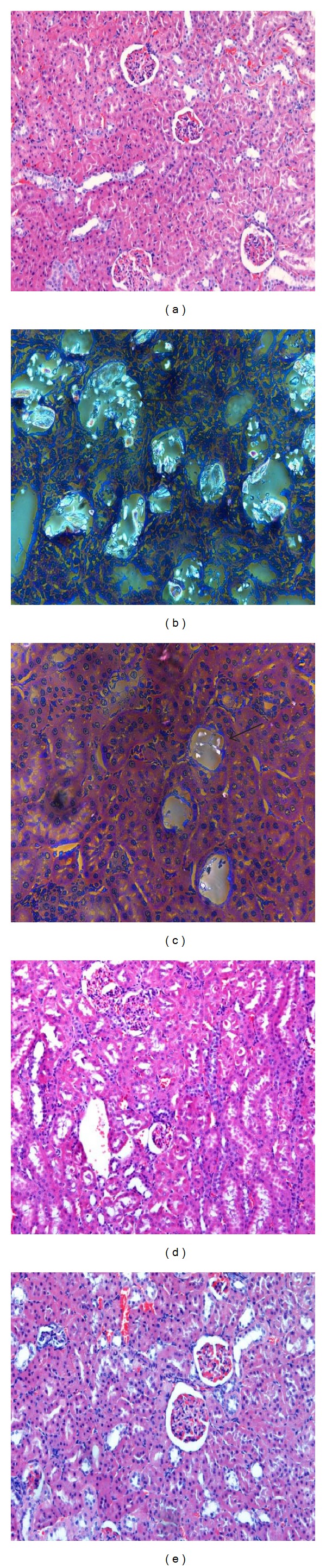
(a) Normal cellular structure of control rats kidney (Gr I). (b) Ethylene-glycol-induced urolithic rats kidney showing irregular crystals dilation and inflammation under polarized microscope (Gr II). (c) Kidneys of urolithic rats treated with *GPAE* (50 mg/kg) showing near normal cellular structure under polarized microscope (Gr III). (d) Kidneys of urolithic rats treated with *GPAE* (100 mg/kg) showing normal cellular Structure (Gr IV). (e) Kidneys of urolithic rats treated with Cystone (100 mg/kg) showing normal cellular Structure (Gr. V). Arrow indicates CaOx crystals.

Histology studies of the kidney regions like cortex, medulla, and papilla in the urolithic animals showed many birefringent crystalline deposits under polarized light microscope (Figure 2(b)). Such deposits were found in 3 out of 6 rats from *GPAE* low dose (Figure 2(c)), 1 out of 6 rats from *GPAE* high dose (Figure 2(d)), and 3 out of 6 rats (Figure 2(e)) from Cystone group but visibly smaller and less abundant compared to urolithic control animals. The tissue morphology and intactness of the kidneys of *GPAE-* and Cystone-treated groups were comparable to the normal control animals (Figure 2(a)). The atomic absorption spectrophotometer analysis revealed decreased Ca content in the *GPAE-* and Cystone- (*P* < 0.05) treated groups as compared with the urolithic control groups wherein the *GPAE* at 100 mg/kg shown significant decrease of Ca (*P* < 0.01) (Table 4).

#### 3.2.3. Lipid Peroxidation Inhibitory Effect

Stone-inducing treatment increased MDA and carbonyl protein content (*P* < 0.01) and decreased GSH level (*P* < 0.05) and activities of the antioxidant enzymes including SOD (*P* < 0.01), GPX (*P* < 0.05), and catalase (*P* < 0.01) in kidneys of the urolithic rats as compared to control animals. Activities of oxalate forming enzymes including GOx (*P* < 0.01) in liver and LDH (*P* < 0.01) in both liver and kidneys were found to be enhanced in urolithic group compared to control animals whereas treatment with *GPAE* was found to be protective against this effect and oxidative changes induced by urolithic treatment at higher doses (Table 4, Figure 1(b)).

## 4. Discussion

Phytotherapy approach is attempted in this study as an alternative to primary healthcare. The *Gokshuradi Polyherbal Formulation* (*GPF*) which is traditionally being utilized in Indian folk medicine since several decades to treat kidney stones was evaluated for its antiurolithic effects. In our earlier studies, we have clinically evaluated the efficacy of this preparation and found its potential in naturally induced urolithic patients [4]. The present study is a reverse pharmacology approach wherein the scientific studies were undertaken under controlled conditions to validate the traditional use of *GPF* and determine its underlying mechanism of action on calcium oxalate urolithiasis.

Calcium oxalate stone development is a multifactorial process involving various etiological factors. Studies have shown that calcium oxalate crystal deposition leads to the cellular injury mediated by lipid peroxidation through free oxygen radical generation. Studies revealed that these cellular injuries favor the events of calcium oxalate retention in renal tubules which is significant for further stone development [33–35]. Recent clinical data are also supporting this finding that formation of urinary stones leads to the oxidative stress in patients [36]. Antioxidants such as vitamin C, catechin, and selenium have proved to be protective against such oxidative injury due to calcium oxalate crystal deposition as evidenced from various experimental studies [37–39]. In this regard, antioxidant potential of *GPAE* was determined by free radical scavenging and lipid peroxidation inhibitory activity to study its mechanism of action. Interestingly, *GPAE* revealed DPPH free radical scavenging and inhibited lipid peroxidation of rat kidney homogenate similar to a standard antioxidant chemical, BHT.

In the diuretic activity study, *GPAE* altered urine output in a dose dependant manner and maximum diuresis observed at 100 mg/kg. At next higher dose that is, 200 mg/kg, it did not lead to any further increase in the urine volume and remained comparable to the normal control group. The disappearance of the diuretic effect of these polyherbal extracts at this higher dose is not completely understood. Increase in urine volume by *GPAE* was also associated with the increase in Na^+^ and K^+^ electrolyte loss similar to the standard diuretic furosemide. Increase in urine volume, thus suggestive of possible loss of these electrolytes leading to this effect. *GPAE*-treated group is also revealed increase in urine pH and decrease in urine Ca^2+^ at 100 mg/kg like that of furosemide-treated groups. These hypocalciuric and diuretic effects of *GPAE* may help to reduce urinary super saturation of calcium salts and thus can prevent further stone formation.

Further antiurolithic effect of *GPAE* was evaluated in an animal model of urolithiasis induced by ethylene glycol and ammonium chloride in combination. This model is frequently used in calcium oxalate stone formation and therefore was selected for this study [25, 40, 41]. After 21 days, administration of EG +AC showed larger and aggregated crystals in urolithic control animals compared to the *GPAE-* and Cystone-treated groups. These calcium oxalate crystal agglomerates get deposited in the renal tubules and further develop into renal stones [25]. *GPAE* reduced the polyuria associated with lithogenic treatment as compared to urolithic control groups. As per the earlier reports, stone induction treatment leads an increase in oxalate and decrease in Ca^2+^ excretion. This is confirmed in the present study in which urolithic control animals showed the similar effect, and *GPAE-* and Cystone-treated groups reversed this effect [42, 43].

Lithogenic treatment resulted in increased calcium oxalate deposition in the urolithic control groups along with oxidative stress determined from increased levels of markers of oxidative damage, that is, MDA and protein carbonyl content, along with decreased activity of antioxidant enzymes and GSH levels in kidneys accompanied with abnormal renal function. The renal tubules were dilated in entire kidneys of all untreated rats leading to disturbed renal flow by large crystal aggregates. However, *GPAE* revealed significant decrease in all these lithogenic markers at higher doses along with the antioxidant activity.

 The present investigation thus reports the significant antiurolithic potential of *GPAE* at higher doses by the combination of antioxidant, diuretic, and hypocalciuric effects. The results of the formulation were found significant as compared with Cystone which is a standard drug available in the market used in the treatment of urolithiasis, and diuretic activity was comparable with the standard diuretic drug, Furosemide.

## 5. Conclusions

The inhibitory effect of *GPAE* on calcium oxalate crystal retention in renal tubules thus can be attributed to its diuretic, hypocalciuric, antioxidant effect along with its potential to inhibit biochemical parameters involved in oxalate metabolism which rationalize its medicinal use for urinary stone disease. The present study will certainly benefit the scientific community worldwide in providing a source for isolation and designing a molecule in this research pipeline.

## Figures and Tables

**Table 1:** Diuretic effect of *Gokshuradi polyherbal aqueous extracts (GPAEs)*.

Groups	Urine vol. (mL)/100 g body weight/6 h	Na^+^ (mmol)/100 g body weight/6 h	K^+^ (mmol)/100 g body weight/6 h	Ca^2+^ (mmol)/100 g body weight/6 h	pH
Control	0.59 ± 0.14	0.09 ± 0.03	0.10 ± 0.02	7.82 ± 1.62	5.87 ± 0.06
*GPAE* 25 mg/kg	1.23 ± 0.21	0.13 ± 0.01	0.15 ± 0.02	6.22 ± 2.29	6.85 ± 0.05
*GPAE* 50 mg/kg	1.51* ± 0.18	0.19 ± 0.01	0.19 ± 0.05	3.69 ± 1.76	6.93 ± 0.18
*GPAE* 100 mg/kg	1.72** ± 0.11	0.26* ± 0.02	0.22* ± 0.03	2.37* ± 1.33	7.32** ± 0.24
Furosemide 10 mg/kg	2.10** ± 0.31	0.38** ± 0.07	0.21** ± 0.02	1.26* ± 0.49	7.39** ± 0.27

Values given are mean ± S.E.M. (*n *= 6).

*P < 0.05.

**P < 0.01 versus control.

**Table 2:** Effect of *GPAE* and standard drug on 24 h urinary biochemical parameters.

Parameters	Control	Urolithic	*GPAE* 50 mg/kg	*GPAE* 100 mg	Standard drug 100 mg/kg
Change in body weight (%)	11.88 ± 1.53	−4.78** ± 1.08	2.32^∗∗, ##^ ± 1.45	8.86^##^ ± 2.06	5.43^∗∗, ##^ ± 1.23
Water intake mL/24 h	6.22 ± 0.39	24.35** ± 7.23	14.3 ± 2.32	8.45^#^ ± 1.17	11.2 ± 2.21
Urine vol. (mL)	5.82 ± 0.45	26.25** ± 6.76	16.43* ± 3.24	10.46^∗, #^ ± 1.26	13.65* ± 2.84
pH	6.38 ± 0.09	6.17 ± 0.16	6.27 ± 0.11	6.45 ± 0.14	6.32 ± 0.15
Oxalate (mg)	0.38 ± 0.10	2.12** ± 0.21	1.35** ± 0.25	0.68^##^ ± 0.11	1.89** ± 0.18
Ca^2+^ (mg)	3.67 ± 0.24	1.84** ± 0.16	2.63* ± 0.29	3.16^#^ ± 0.64	2.89* ± 0.41
Mg^2+^ (mg)	3.28 ± 0.32	2.67 ± 0.11	3.42 ± 0.24	3.76^#^ ± 0.31	2.87 ± 0.32
Citrate (mg)	23.10 ± 1.21	18.5 ± 1.90	19.12 ± 2.63	19.31 ± 1.22	18.34 ± 2.54
IP^a^ (mg)	6.26 ± 0.82	7.89 ± 1.25	7.57 ± 0.55	7.55 ± 0.75	7.34 ± 0.32
UA^b^ (mg)	0.58 ± 0.17	1.23 ± 0.40	1.05 ± 0.25	0.83 ± 0.22	0.98 ± 0.22

Values given are mean ± S.E.M. (*n *= 6).

*P < 0.05.

**P < 0.01 versus control group.

^#^ *P* < 0.05.

^##^ *P* < 0.01 versus urolithic group.

^a^Inorganic phosphate. ^b^Uric acid.

**Table 3:** Effect of *GPAE* and standard drug on serum parameters.

Parameters	Control	Urolithic	*GPAE* 50 mg/kg	*GPAE* 100 mg	Standard drug 100 mg/kg
SC^c^ (mg/dL)	0.86 ± 0.05	1.43** ± 0.12	1.26 ± 0.16	0.89^#^ ± 0.10	1.08 ± 0.12
CC^d^ (mg/min)	0.86 ± 0.06	0.50** ± 0.06	0.64 ± 0.10	0.85^##^ ± 0.07	0.72 ± 0.12
BUN^e^ (mg/dL)	19.89 ± 1.40	57.04** ± 12.46	46.3 ± 11.40	20.5^##^ ± 1.08	36.2 ± 10.20

Values given are mean ± S.E.M. (*n *= 6).

*P < 0.05.

**P < 0.01 versus control group.

^#^ *P* < 0.05.

^##^ *P* < 0.01 versus urolithic group.

^c^Serum creatinine. ^d^Creatinine clearance. ^e^Blood urea nitrogen.

**Table 4:** Effect of *GPAE* and standard drug on kidney and liver parameters.

Parameters	Control	Urolithic	*GPAE* 50 mg/kg	*GPAE* 100 mg	Standard drug 100 mg/kg
Kidney weight (g)	0.78 ± 0.05	1.21** ± 0.12	1.09* ± 0.14	0.82^#^ ± 0.07	0.98* ± 0.12
CD^f^	Absent	Significant	Moderate	Not significant	Moderate
MDA^g^ (nmol)	0.64 ± 0.14	6.52** ± 0.85	3.06 ± 1.10	1.09^##^ ± 0.42	2.85 ± 1.08
PCC^h^ (nmol)	5.08 ± 0.68	13.04** ± 1.36	10.12 ± 1.88	8.05^#^ ± 1.14	9.80 ± 1.23
GSH^i^ (nmol)	17.89 ± 0.98	13.10* ± 1.33	15.03 ± 1.82	16.98^#^ ± 1.57	14.02 ± 1.31
SOD^j^ (U)	6.22 ± 0.84	3.01** ± 0.29	4.58 ± 0.32	5.48^#^ ± 0.56	4.21 ± 0.21
GPX^k^ (U)	0.58 ± 0.05	0.32* ± 0.04	0.47 ± 0.08	0.53^#^ ± 0.07	0.48 ± 0.05
Cat^l^ (*μ*MH_2_O_2_/min)	32.89 ± 2.55	16.89** ± 1.98	20.92 ± 5.11	24.90 ± 2.32	21.08 ± 4.32
GOx^m^ (nmol/mg)	1.61 ± 0.07	2.35 ± 0.06**	1.77 ± 0.17	1.50 ± 0.10^#^	1.65 ± 0.17
LDH^n^ (U/mg) (Liver)	1.54 ± 0.06	4.03 ± 0.09**	1.49 ± 1.08	1.51 ± 0.06^#^	1.46 ± 1.04
LDH^o^ (U/mg) (Kidney)	2.82 ± 0.16	4.68 ± 0.19**	3.19 ± 1.08	3.07 ± 1.08^#^	3.32 ± 1.04
Calcium^p^ (mg/g) (Kidney)	0.292 ± 0.16	0.498 ± 0.082	0.375 ± 0.078	0.316 ± 0.021^##^	0.369 ± 0.038^#^

Values given are mean ± S.E.M. (*n *= 6).*P < 0.05. **P < 0.01 versus control group. ^#^ *P* < 0.05. ^##^ *P* < 0.01 versus urolithic group.

^f^Crystal deposits/100x: field, ^g^malondialdehyde, ^h^protein carbonyl content,^ i^reduced glutathione, ^j^superoxide dismutase, ^k^glutathione peroxidase, ^l^catalase, ^m^glycolate oxidase (nmol of glyoxylate formed/mg protein), ^n^liver lactate dehydrogenase (U/mg protein), ^o^kidney lactate dehydrogenase (U/mg protein), and ^p^kidney calcium content.
